# Interactive Effect of Cultivars, Crop Years and Rootstocks on the Biochemical Traits of *Prunus persica* (L.) Batsch Fruits

**DOI:** 10.3390/plants12122325

**Published:** 2023-06-15

**Authors:** Roberto Ciccoritti, Rossella Manganiello, Francesca Antonucci, Danilo Ceccarelli

**Affiliations:** 1 Consiglio per la ricerca in agricoltura e l’analisi dell’economia agraria (CREA)—Centro di ricerca Olivicoltura, Frutticoltura e Agrumicoltura—Via di Fioranello 52, 00134 Rome, Italy; roberto.ciccoritti@crea.gov.it (R.C.); danilo.ceccarelli@crea.gov.it (D.C.); 2 Consiglio per la ricerca in agricoltura e l’analisi dell’economia agraria (CREA)—Centro di ricerca Ingegneria e Trasformazioni agroalimentari—Via della Pascolare 16, Monterotondo, 00015 Rome, Italy; rossella.manganiello@crea.gov.it

**Keywords:** climatic effects, phytochemicals, multivariate statistics, nutraceutical plant profile, grafting

## Abstract

Peach fruit is one of the most economically widespread temperate fruits, whose productivity, and nutritional and sensory qualities are determined by interactions among several environmental and genetic factors, rootstocks, agronomic practices and pedo-climatic conditions. In recent years, climate change has prompted peach breeding programs to use specific rootstocks that are well adapted to unusual soil and climate characteristics, thus improving the plant’s adaptability and fruit quality. The aim of this work was to assess the biochemical and nutraceutical profile of two different peach cultivars, considering their growth on different rootstocks over three crop years. An analysis was carried out evaluating the interactive effect of all factors (i.e., cultivars, crop years and rootstocks) revealing the advantages or disadvantages on growth of the different rootstocks. Soluble solids content, titratable acidity, total polyphenols, total monomeric anthocyanins and antioxidant activity in fruit skin and pulp were analyzed. An analysis of variance was performed to assess the differences between the two cultivars considering the effect of rootstock (one way) and crop years, rootstocks and their interaction (two ways). In addition, two principal component analyses were performed separately on the phytochemical traits of the two cultivars to visualize the distributions of the five peach rootstocks during the three crop years. The results showed that fruit quality parameters are strongly dependent on cultivars, rootstocks and climatic conditions. All these aspects could be useful for the choice of rootstock in relation to agronomic management, making this study a valuable tool for choosing the best rootstock, considering simultaneously more factors affecting peaches’ biochemical and nutraceutical profile.

## 1. Introduction

Peach [*Prunus persica* (L.) Batsch] fruits represent one of the most widespread stone fruits, due to its good values from an energetic dietetic, nutritional and nutraceutical point of view. The peach fruit represents an important source of antioxidants, especially phenols, vitamin C and carotenoids, which are present in greater quantities, especially in the peel, although this part is not appreciated by consumers [1,2]. The antioxidant capacity due to the presence of phenolic compounds in these fruits is strongly influenced by the genotype [3]. In recent years, the consumption of peaches has decreased significantly globally, mainly due to dissatisfaction among consumers, who find fruits on the market to be mostly tasteless and of poor consistency, as they are harvested before they are fully ripe so that they can have a longer shelf-life. The main field factors that can influence fruit quality are the genotype, rootstock, orchard cultivation systems, harvest time and agro-climatic conditions. The lower quality is also due to bad post-harvest handling and storage [2].

From this perspective, peach and nectarine breeding programs are developing new genotypes that meet consumer expectations, and are trying to strike the right balance between quality and maturity at market harvest time. In addition, climatic changes in recent years have prompted peach breeding programs to improve the adaptability of peach trees to different soil and climatic conditions and cultivation systems to support high production standards, increase consumption and maintain a sustainable and profitable industry. For example, by using specific rootstocks that are adapted to unusual soil or water stress characteristics, fruit quality can be improved while also expanding ripening seasons [4]. In general, rootstocks influence many vegetative and reproductive traits of plants, including tree size, water and mineral requirements, climatic adaptation of flower buds, flowering and ripening times, yield and fruit quality [5]. The nutritional quality of the fruits is closely related to the interaction of the rootstock with water and nutrient availability in the soil [6]. Rootstocks provide a cultural tool for peach growers to increase productivity and improve efficiency via better tree survival, controlled tree vigor and increased fruit size, yield and quality. Thus, the choice of rootstock becomes as economically important as the scion cultivar whenever peach trees must be grown on soils with high bulk density, coarse texture (sand), parasitic nematodes, root rot fungal pathogens, high pH or other orchard replant problems [7].

Weather and climatic conditions and the growing environment, such as, for example, canopy irradiation, vigor management and carbon supply, also strongly influence fruit quality, especially exocarp metabolic profiles [8]. High CO_2_ concentration, high temperature and limited water availability, as a result of climate change, have a negative impact on flowering and production [9].

In this scenario, the aim of the present study was to assess the biochemical and nutraceutical profile of two different peach cultivars, considering their growth on different rootstocks over three crop years. This analysis was carried out by evaluating the interactive effect of all factors (i.e., cultivars, crop years and rootstocks), revealing the advantages or disadvantages about growth of the different rootstocks.

## 2. Materials and Methods

### 2.1. Plant Material and Climatic Data Collection

This study was performed during three crop years (2011, 2017 and 2019) at the experimental farm of Centro di ricerca Olivicoltura, Frutticoltura e Agrumicoltura of Rome—Consiglio per la ricerca in agricoltura e l’analisi dell’economia agraria (CREA) (Central Italy, 41.8000° N, 12.5690° E, alt. 86 m a.s.l.).

Two different peach cultivars, grafted on five different rootstocks, grown with the same agronomic techniques (i.e., fertilization, irrigation and pest control), were considered. For the chemical and pomological analyses, fruits were harvested at consumption maturity [10].

The *P. persica* cultivars used were Ghiaccio-1*, characterized by their total lack of pigment and their white pulp, fruit with long storage capability, and sweet and aromatic test coupled to a rustic tree, and Romestar*, characterized by deep yellow pigmented fruit pulp with red pigment around the stone [11]. Finally, rootstocks (i.e., GF677, Cadaman^®^ Avimag, Barrier^®^, Isthara^®^ and GxN22 (Felinem)) were chosen in relation to the different characteristics, as reported in Table 1.

Climatic data (i.e., daily mean temperature, daily mean rainfall, daily mean relative humidity and the average total hourly solar radiation) during the three years were acquired from the Agenzia Regionale per lo Sviluppo e l’Innovazione dell’Agricoltura del Lazio (ARSIAL) weather station (RM17SIE) positioned at Marino (Rome).

Three sets of samples were collected from each cultivar (i.e., Ghiaccio-1* and Romestar*) for each rootstock for each of the three years. For the quality traits analyses, three replicates of fresh pitted fruits (about 1.5 kg) from each set were randomly sampled. For phytochemical traits determination, the samples were stored at −80 °C until the analyses.

### 2.2. Chemicals

All reagents were of analytical High-Performance Liquid Chromatography (HPLC)-grade (Merk Life Science S.r.l, Milan, Italy). Folin–Ciocalteau reagent, 2,2-diphenyl-1-picrylhydrazyl (DPPH), (±)-6-hydroxy-2,5,7,8-tetramethylchromane-2-carboxylic acid (commonly called Trolox) (T), sodium carbonate and gallic acid (GA) and cyanidin 3-O-glucoside (CG) were purchased from Sigma-Aldrich (St. Louis, MO, USA). Milli-Q water (Millipore, Bedford, MA, USA) passed through 0.45 nylon membrane filters (Pall Corporation, Ann Arbor, MI, USA) was used for the study.

### 2.3. Analytical Methods

#### 2.3.1. Pomological Traits Determination

Pitted fresh fruits (about 500 g per sample) were homogenized, and titratable acidity (TA) and pH were determined, according to Ceccarelli et al. [10], on 10 g aliquots diluted to 50 mL with distilled water using an automatic titration system (785 DPM Titrino, Metrohm Ldt, Herisau, Switzerland). TA content was expressed as mEq L^−1^ of NaOH 0.1 M. Soluble solids content (SSC) was determined on fruit juice with a digital refractometer (Refracto 30PX, Mettler Toledo, Greifensee, Switzerland) and expressed as g 100 g^−1^ FW (Brix degrees).

#### 2.3.2. Phytochemical Content, Total Monomeric Anthocyanin and Antioxidant Activity Extraction

Defrosted samples (about 5 g) were homogenized with a blender (Ultra-Turrax T25, IKA Labortechnik, Staufen, Germany) in 25 mL of methanol solution (methanol/water 70/30 *v*/*v*), adding 5 mM HCl to determine total phenolic content (TPC), total monomeric anthocyanin (TMA) and antioxidant activity (AA). According to Ceccarelli et al. [10], the extraction was carried out under shaking in a thermostatic bath at 37 °C for 2 h, and then were centrifuged (centrifuge mod. 4239R, ALC International—Milan, Italy) at 8000× *g* for 15 min at 5 °C, recovering the supernatant.

#### 2.3.3. Total Phenolic Content Determination

The TPC was determined using the Folin–Ciocalteu method as reported by Ceccarelli et al. [10]. Results were calculated and expressed in mg of gallic acid equivalent (GAE) 100 g^−1^ FW. Determination of GAE was performed using the GA standard curve (0.025–0.5 mg mL^−1^). Briefly, 0.4 mL of extract, were added to 16.0 mL water, 2.0 mL of Folin–Ciocalteu phenol reagent and 6.0 mL of 1M sodium carbonate, and the final volume was adjusted to 25 mL with the same solution used for the extraction. Samples were read at 760 nm after 2 h using an Evolution 300 UV–Vis Spectrophotometer (Thermo Electron Scientific Instruments, Madison, WI, USA). The samples were analyzed in triplicate.

#### 2.3.4. Total Monomeric Anthocyanins Determination

TMA were determined using the pH differential method as described by Giusti and Wrolstad [15], using the UV–Vis spectrophotometer at 510 and 700 nm. Results were calculated and expressed as mg cyanidin 3-O-glucoside equivalents (CGE) 100 g^−1^ FW. All samples were analyzed in triplicate.

#### 2.3.5. Antioxidant Activity Evaluation

The AA of the extracts was determined using the DPPH (2,2-diphenyl-1-picrylhydrazyl) method as described by Ceccarelli et al. [10]. In this procedure, 1.5 mL of DPPH solution was added to 1.5 mL of fruit extract, and after 15 min, absorbance at 515 nm was determined using the UV–Vis Spectrophotometer. The percent inhibition activity of fruit extract was calculated as:

[(A_0_ − A_1_)/A_0_]⋅100

where A_0_ was the control absorbance and A_1_ the extract absorbance. Trolox (0.5–10 μg mL^−1^) was used as the reference compound and AA was expressed as μg of Trolox equivalent (TE) mg^−1^ FW. All samples were analyzed in triplicate.

### 2.4. Statistical Analysis

All the statistical analyses described below were carried out on the mean of three replicates for each cultivar. In addition, all the rootstocks were considered for the analyses (Past v. 4.02). Firstly, a one-way analysis of variance (ANOVA) was performed on the whole dataset to evaluate the differences between the two cultivars (i.e., Ghiaccio-1* and Romestar*) considering the effect of rootstock on phytochemical composition. Then, a two-way ANOVA considering the effects of crop years (Y), rootstock (R) and their interaction (Y × R), followed by a post hoc Tukey test, was carried out. In addition, two Principal Component Analyses (PCA) were performed (considering the two cultivars separately) on the mean data of SSC, TA, TPC, TMA and AA to visualize the distribution of the five peach rootstocks during the three crop years.

## 3. Results

### 3.1. Climatic Condition

**Figure 1:**
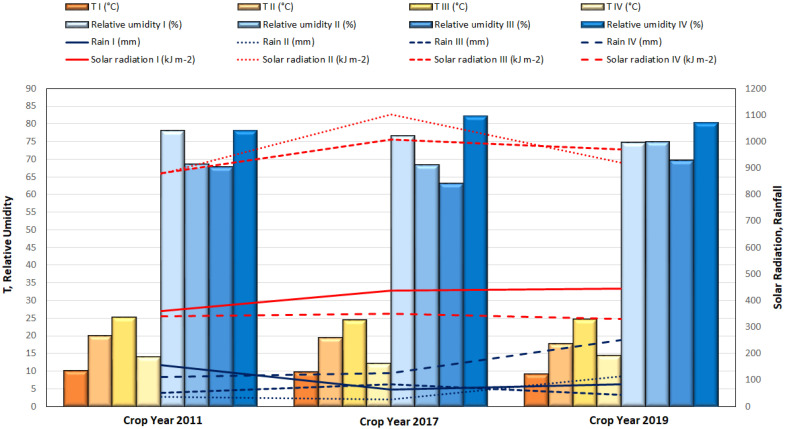
Means of the daily climatic data (i.e., air temperature at 2 m (T, °C), relative humidity at 2 m (%), rainfall (mm) and solar radiation (kJ m^−2^) during the four trimesters of the 2011, 2017 and 2019 crop years. The first quarter (I) refers to the months of January to March, the second (II) to the months of April to June, the third (III) to the months of July to September and the fourth (IV) to the months of October to December.

The hourly variation in weather and climate parameters (i.e., average daily temperature, average daily precipitation, average daily relative humidity and average total solar radiation) recorded during the experimental crop years (2011, 2017 and 2019) is shown in Figure 1.

The monthly average air temperature ranged from 6.0 °C (January 2017) to 27.4 °C (August 2017). The highest average temperature was recorded in August 2017 (28.2 °C), and the lowest in January 2017 (5.5 °C). In winter, minimum temperatures never fell below 5 °C, while maximum temperatures in summer averaged around 25–26 °C. The average air temperature during the growing season (II trimester) was around 19 °C for the 2011–2017 crop years and 17.6 °C for 2019. The average solar radiation was very high in the second and third quarters of 2017, reaching a maximum value of 1240 kJ m^−2^ in June, while lower and similar values were reported during the 2011 and 2019 crop years. The rainfall distribution during the growing season was higher in 2019; in particular, May 2019 was an extremely rainy month (average value 235 mm), while it never rained in the following month. In the same period of the 2011 and 2017 crop years, the average total amount of rainfall was very low, varying between 30 and 80 mm. Finally, the highest average monthly relative humidity was recorded in November 2019 (86.7%), corresponding to the maximum rainfall (516 mm), while the lowest average relative humidity was recorded in July 2017 (51.6%).

### 3.2. Peach Cultivars Biochemical Variability

**Figure 2:**
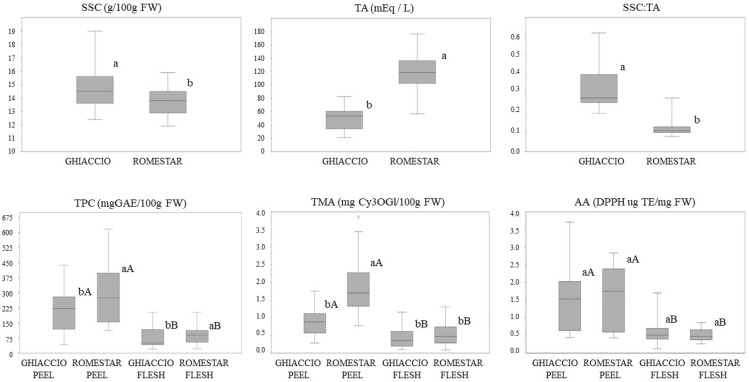
Box plots extracted from the one-way analysis of variance (ANOVA) examining soluble solid content (SSC), titratable acidity (TA), soluble solid content and titratable acidity ratio (SSC: TA) of the entire pitted fresh fruits, and the total phenolic compound (TPC), total monomeric anthocyanins (TMA) and antioxidant activity (AA) of the peel and flesh of the two cultivars Ghiaccio-1* (Ghiaccio) and Romestar* during the three crop years 2011, 2017 and 2019. Values belonging to the same traits without letters in common are statistically different according to LSD (*p* ≤ 0.05). Lower-case letters are used for intragroup difference evaluation, upper-case letters are applied for intergroup evaluation (peel vs. flesh).

The one-way ANOVA to evaluate the rootstock effect between the two cultivars (i.e., Ghiaccio-1* and Romestar*) showed that the two cultivars were significantly (*p* < 0.05) different for all investigated parameters, except for antioxidant activity. Figure 2 shows the box plots displaying these values.

For Ghiaccio-1*, the SSC and the TA were higher than those of Romestar*, when considering all the five different rootstocks together (15 ± 1 g/100 g FW and 50 ± 10 mEq L^−1^, respectively). Considering the peel bioactive compounds (TPC and TMA), the highest values were observed in Romestar*, whereas no significant differences were found in AA (Figure 2). No significant differences among TPC, TMA or AA were found between the two cultivars in flesh fruits.

### 3.3. Peach Years and Rootstock Biochemical Variability

The results of the two-way ANOVA reported in Table 2 show significant effects (*p* < 0.001) of crop years (Y), rootstock (R) and their interaction (Y × R) for all investigated phytochemicals in Ghiaccio-1*.

Y (Table 2A) was the main factor that affected all parameters, except for flesh AA, which was strongly influenced by the interaction Y × R. In particular, the highest TA value values of the Y mean data were observed in 2017 and 2019(61 ± 12 and 55 ± 5 mEq L^−1^, respectively), while the lowest was detected in 2011 (32 ± 6 mEq L^−1^).

A low TA variability was observed among the rootstocks, ranging from 54 ± 6 (GxN22) to 46 ± 5 mEq L^−1^ in the Isthara^®^. The highest SSC amount was found in 2017, ranging from 14.1 to 17.9 g 100 g^−1^ FW (Table 2B). Among the rootstocks, the mean highest SSC was found in GxN22 (mean 16 ± 1 g 100 g^−1^ FW), followed by Barrier^®^ (mean 15 ± 1 g 100 g^−1^ FW) and by Cadaman^®^ and Isthara^®^ (14.2 ± 0.9 g 100 g^−1^ FW). In addition, slight differences in SSC:TA ratio were found among Isthara^®^ and the others (Table 2B).

Significant differences (*p* < 0.001) were observed among the Y and between the two cultivars, showing the highest TPC in 2017 both in peel and flesh (307 ± 80 and 142 ± 36 mg GAE 100 g^−1^ FW, respectively). The highest TMA values were found in peel (1.2 ± 0.3 mg Cy3OGl 100 g^−1^ FW) in 2017 and in flesh in 2019 (2.2 ± 0.8 mg Cy3OGl 100 g^−1^ FW; Table 2B). Finally, the mean highest AA in peel was found in 2011, whereas no significant differences were found among the years in flesh.

Among the studied rootstocks, Barrier^®^, Cadaman^®^ and Isthara^®^ showed an interesting phytochemical profile characterized by the highest TPC, TMA and AA values. The rootstocks GxN22 and GF677 showed low TPC, TMA and AA values both in peel and flesh (Table 2B).

Regarding Romestar*, the two-way ANOVA revealed similar effects of Y, R and Y × R for all the parameters. Additionally, for this cultivar, the Y was the main discriminant factor except for SSC and SSC:TA ratio, which were strongly affected by Y × R (Table 3A).

In particular, for 2017 and 2019, the highest TA values were equal to 125 ± 24 and 128 ± 16 mEq L^−1^, respectively, while the lowest was observed for 2011 (96 ± 21 mEq L^−1^; Table 3B).

High TA variability was observed among the rootstocks, which ranged from 131 ± 10 for GF677 to 96 ± 15 mEq L^−1^ for Isthara^®^. The highest SSC was found in 2017, ranging from 13.2 to 15.2 g 100 g^−1^ FW (Table 2B). Among the rootstocks, the highest mean SSC was found in Isthara^®^ (14.5 ± 0.9 g 100 g^−1^ FW), followed by Barrier^®^ (14.3 ± 0.8 g 100 g^−1^ FW) and by Cadaman^®^ and GxN22 (13.2 ± 0.9 g 100 g^−1^ FW). Finally, slight differences in SSC:TA ratio were found between Isthara^®^ and all the others (Table 3B).

The years 2019 and 2017 showed the highest peel values of TPC, TMA and AA, whereas the lowest were observed in 2011 (Table 3B). The flesh showed the highest value of TPC (144 ± 37 mg GAE 100 g^−1^ FW) during 2017, and of AA during 2011 (0.7 ± 0.1 µg g^−1^ TE FW).

As regards rootstocks, only the TPC values showed significant differences. In detail, Isthara^®^ showed the highest TPC value both in its peel and flesh.

### 3.4. Chemometric Elaboration

**Figure 3:**
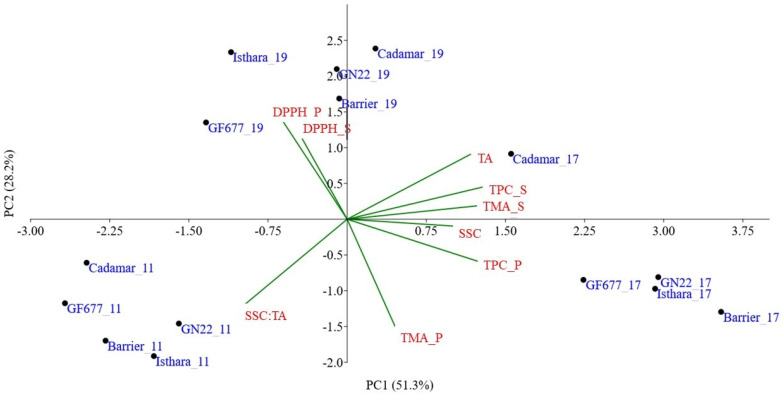
Principal Component Analysis (PC1 vs. PC2) of Ghiaccio-1* samples performed on the five rootstocks (i.e., Cadaman^®^, GF677, GxN22, Isthara^®^ and Barrier^®^) analyzed in 2019 (black dots), 2017 (red dots) and 2011 (blue dots). The scatter plot reports: solid soluble content (SSC), titratable acidity (TA), total phenolic content (TPC), total monomeric anthocyanins (TMA) and antioxidant activity (AA) in peel (S) and flesh (P).

To visualize the distribution of the five peach rootstocks during the three crop years according to the phytochemical traits (i.e., SSC, TA, TPC, TMA and AA), two separate PCAs were performed for the two cultivars. Figure 3 shows the scatter plot of Ghiaccio-1* considering the five different rootstocks. The first two principal components (PC1 and PC2) represent an explained variance equal to 58.3% and 28.3%, respectively. The PC1 was positively correlated with SSC, TPC and TMA. Meanwhile, the PC2 was positively correlated with AA and TA. It is possible to observe well-defined clusters grouping the samples in three main classes (Figure 3). The first one was placed in the first and fourth quadrant and included all samples grown during 2017, characterized by the highest SSC, TPC and TMA values. The second one was located in the first and second quadrant, and included all the samples grown during 2019, and was characterized by the highest AA. Finally, the third group was located in the third quadrant, and presented the highest SSC:TA ratio values.

**Figure 4:**
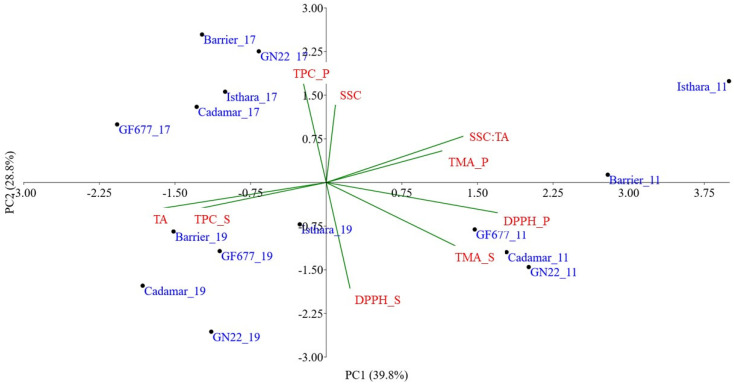
Principal Component Analysis (PC1 vs. PC2) of Romestar* samples performed on the five rootstocks (i.e., Cadaman^®^, GF677, GxN22, Isthara^®^ and Barrier^®^) analyzed in 2019 (black dots), 2017 (red dots) and 2011 (blue dots). The scatter plot reports: solid soluble content (SSC), titratable acidity (TA), total phenolic content (TPC), total monomeric anthocyanins (TMA) and antioxidant activity (AA) in peel (S) and flesh (P).

The PC1 in Figure 4 was positively correlated with TMA, SSC:TA ratio and AA, while the PC2 was positively correlated with TPC and SSC. Additionally, for this cultivar, the PCA reported three well-defined clusters representing the three crop years. In each group, the samples were spread in relation to the rootstocks, which influenced the fruits’ biochemical composition.

## 4. Discussion

The aim of this study was the valuation of the advantages of five different rootstocks through the characterization of the biochemical and nutraceutical profiles of two different peach cultivars (i.e., Ghiaccio-1* and Romestar*). Several authors [4,16,17,18,19] have underlined the key role of rootstock in determining the quality of production and the nutraceutical characteristics of fruits. Numerous of these specified that some peach rootstocks increased the yield, size and quality of commercial peach [5,8,16,20,21]. To strengthen the purpose of this research, three crop years were considered. This, coupled with chemometric elaboration, allowed the evaluation of the effects of climatic conditions on the fruits’ qualitative traits, defining the effects of rootstock during the experiment. Generally, as reported in this work, the fruit quality parameters were found to be strongly dependent on the cultivars, rootstocks and climatic conditions (Figure 1; Table 2 and Table 3). This has been confirmed by many studies present in the literature [9,16,22,23,24].

As reported in Table 2 and Table 3, Ghiaccio-1* showed the highest SSC and the lowest TA values with respect to Romestar*. Its high SCC:TA ratio, its acid levels and its soluble solid concentration could make it very suitable for consumers [25,26]. Generally, significant differences in TPC and TMA were found in this study between the two cultivars with respect to AA, probably due to their specific chemical composition.

The multivariate statistical PCA was applied to evaluate the similarity of phytochemical parameters between the different rootstock types during the three crop years. Figure 3 shows that, for Ghiaccio-1*, Cadaman^®^ rootstock was located the furthest from the others. All the samples grafted on Cadaman^®^ were located in the positive side of PC2, highlighting high values of TA and AA, peel TPC and TMA, and suggesting high adaptability to the environment. Finally, the Barrier^®^ rootstock appears to induce higher sweetness, low acidity and higher TPC content in Ghiaccio-1*.

On the other hand, Figure 4 shows that, for Romeestar*, the best performance in terms of quality biochemical traits was obtained by using the Isthara^®^ rootstock.

In addition, climatic conditions affect peach quality parameters and chemical composition differently. Moreover, they could influence the phytochemical profile in different ways. For example, water stress in the final stages of growth in plum fruits causes a significant decrease in size, but accelerates maturation and SSC level [27]. Higher precipitation has been found to be related to higher TPC values, as was observed for Romestar* in 2019. The high humidity registered during harvest time in this year could be an additional factor contributing to higher phenols content, possibly due to minor abiotic stress which could have influenced transpiration and photosynthetic activities [28]. In addition, the abundant solar radiation in 2017 contributed to an increase in the amount of the bioactive compounds TPC and TMA in Ghiaccio-1*, thanks to the greater development of pigments, especially in the peel. This was in agreement with the study of Solovchenko and Schmitz-Eiberger [29], which claimed that specific spectral light properties of solar radiation and temperatures are important for the regulation of antioxidant biosynthesis. This research highlighted that the parameter most influenced by climatic conditions is the cultivar.

The results of this work demonstrated that the main bioactive compounds and antioxidant activity are significantly influenced by rootstock, even if it is not possible to define a regular trend. Indeed, rootstocks of similar vigor (Table 1) produced fruits with very different nutritional characteristics, indicating that the effects of climatic condition and grafted varieties could strongly affect the peach quality, underlining the fact that biochemical composition is strictly related to the interaction of rootstock water composition and nutrient soil availability [30].

## 5. Conclusions

Peach fruit quality is highly dependent on cultivars and growing years. Moreover, this is closely related to the choice of rootstock on which the plants are grafted, which is becoming an important parameter to consider in new plantings. In fact, rootstocks are valuable tools used by growers to improve the efficiency of cultivation, such as by increasing plant survival under different soil and climate conditions. In addition, it can control their vigor and increase productivity, with effects on fruit yield and phytochemical characteristics. All these aspects could be useful for the choice of rootstock in relation to the agronomic management, making this study a valuable tool for choosing the best rootstock while simultaneously considering more factors affecting the fruit’s qualitative parameters.

## Figures and Tables

**Table 1:** Rootstocks origin and agronomic characteristics. Modified from [12,13,14].

Rootstocks	Origin	Characteristics
GF677	*Prunus persica* (L.) Batsch x *Prunus amygdalus* Batsch	Induces high tree vigor, rapid entry into production, high production yields (both dry and irrigated). Superior adaptability even to difficult soils, tolerating active lime levels up to 12%. Tolerates water deficiency and stumpiness fairly well. Poor resistance to Agrobacterium, nematodes and Armillaria.
Cadaman^®^ Avimag	*Prunus davidiana* (Carrière) Franch. x *Prunus persica* (L.) Batsch	Medium-to-high vigor (similar to GF677) with good growth rate. Suitable for fresh, poor and even tendentially asphyctic soils. Induces slight earliness of maturity and increase in size. Resistant to some types of nematodes. Presents some polloniferous aptitude.
Barrier^®^	*Prunus davidiana* (Carrière) Franch. x *Prunus persica* (L.) Batsch	Slightly lower vigor than GF677. Good anchorage, good-to-high productivity, induces better fruit size and coloration, slightly delays both flowering and ripening. Suitable for replanting. Resistant to some nematodes, asphyctic soils and chlorosants.
Isthara^®^	(*Prunus cerasifera* Ehrh. x *Prunus salicina* Lindl.) x (*Prunus cerasifera* Ehrh. x *Prunus persica* (L.) Batsch)	Medium vigor, with lower development than “franco” seedling rootstocks, but with good vegetative renewal and decent adaptability. Moderate suckering activity. Induces early fruiting, high yields and good-sized fruit. It is susceptible to Armillaria, but resistant to some nematodes.
GxN22 (Felinem)	*Prunus amygdalus* Batsch x *Prunus persica* (L.) Batsch	In grafted cultivars induces vigor and productivity similar to GF-677 or Hansen 536. High resistance to the main root nematode species that attack *Prunus*. Adapts well to calciferous soil. Resistant toward chlorosis.

**Table 2:** Results of the two-way analysis of variance (ANOVA) of pitted fruit, flesh and peel of the Ghiaccio-1* cultivar: (**A**) two-way ANOVA performed on the mean squares of the five rootstocks (R) for three crop years (Y); (**B**) mean values, for R and Y, of titratable acidity (TA), soluble solids content (SSC), SSC:TA, total phenolic content (TPC), total monomeric anthocyanins (TMA) and antioxidant activity (AA). ^1^ Degrees of freedom (DF); * significant (*p* < 0.05); ** significant (*p* < 0.001). Different letters in the same column indicate significant differences.

Ghiaccio-1*											
	(A)		Pitted Fruit			Peel			Flesh		
	Source	D.F. ^1^	TA	SSC	SSC:TA	TPC	TMA	AA	TPC	TMA	AA
	Crop Years (Y)	2	15,254.1 **	79.32 **	0.68 **	776,527 **	7.7 **	25.5 **	201,693 **	2.7 **	0.96 **
	Rootstock (R)	4	661.6 **	28.67 **	0.02 ns	70,126.8 **	1.5 **	22.5 **	11,088 **	0.7 **	1.66 **
	Y × R	5	3210.3 *	64.22 **	0.03 ns	130,864 **	2.1 **	15.2 **	11,878 **	0.8 **	6.91 **
	Within	75	42.8 ns	44.56 ns	0.19 ns	62,892 ns	0.9 ns	4.5 ns	13,910 ns	1.6 ns	0.52 ns
	Total	89	19,877.4 ns	219.79 ns	0.92 ns	1.1 × 10^6^ ns	12.2 ns	67.6 ns	238,570 ns	5.8 ns	10.05 ns
(B)			TA	SSC	SSC:TA	TPC	TMA	AA	TPC	TMA	AA
ROOTSTOCKS	Barrier^®^		48 ± 8 ab	15.0 ± 1.1 ab	0.4 ± 0.1 a	235 ± 27 a	1.0 ± 0.1 a	1.4 ± 0.1 bc	96 ± 17 a	0.5 ± 0.1 a	0.5 ± 0.2 a
	Cadaman^®^		51 ± 7 a	14.2 ± 0.9 b	0.3 ± 0.1 a	223 ± 20 a	0.9 ± 0.1 a	2.5 ± 0.2 a	76 ± 16 ab	0.3 ± 0.1 b	0.6 ± 0.1 a
	GF677		48.0 ± 6 ab	14.8 ± 1.0 ab	0.3 ± 0.1 a	155 ± 17 b	0.7 ± 0.1 b	1.2 ± 0.1 c	67 ± 10 b	0.3 ± 0.1 b	0.5 ± 0.2 a
	GXN22		54 ± 6 a	15.9 ± 1.3 a	0.3 ± 0.1 a	204 ± 26 ab	0.9 ± 0.2 ab	1.2 ± 0.1 c	68 ± 8 b	0.4 ± 0.1 ab	0.5 ± 0.2 a
	Isthara^®^		46 ± 5 b	14.2 ± 0.8 b	0.3 ± 0.1 a	219 ± 12 a	1.0 ± 0.2 a	1.6 ± 0.2 b	88 ± 12 a	0.6 ± 0.2 a	0.6 ± 0.1 a
			TA	SSC	SSC:TA	TPC	TMA	AA	TPC	TMA	AA
YEARS	2011		31.5 ± 5.5 b	13.8 ± 0.8 bc	0.45 ± 0.08 a	90 ± 27 b	0.6 ± 0.1 b	1.5 ± 0.4 a	43.8 ± 12.0 b	0.5 ± 0.3 b	0.5 ± 0.1 a
	2017		60.8 ± 11.9 a	16.0 ± 1.9 a	0.27 ± 0.05 b	307 ± 80 a	1.2 ± 0.3 a	1.0 ± 0.1 b	142 ± 36 a	0.6 ± 0.1 b	0.6 ± 0.2 a
	2019		55.4 ± 5.1 a	14.6 ± 0.9 b	0.26 ± 0.03 b	223 ± 66 ab	0.9 ± 0.3 ab	0.22 ± 0.08 c	49 ± 20 b	2.2 ± 0.8 a	0.7 ± 0.1 a

**Table 3:** Results of the two-way analysis of variance (ANOVA) of pitted fruit, flesh and peel of the Romestar* cultivar: (**A**) two-way analysis of variance (ANOVA) performed on the mean squares of the five rootstocks (R) for three crop years (Y); (**B**) mean values, for the R and Y, of titratable acidity (TA), soluble solids content (SSC), SSC:TA, total phenolic content (TPC), total monomeric anthocyanins (TMA) and antioxidant activity (AA). ^1^ Degrees of freedom (DF); * significant (*p* < 0.001). Different letters in the same column indicate significant differences.

Romestar*											
(A)			Pitted Fruits			Peel			Flesh		
	Source	D.F. ^1^	TA	SSC	SSC:TA	TPC	TMA	AA	TPC	TMA	AA
	Crop Years (Y)	2	20,291.8 *	6.9 *	0.04 *	1.1 × 10^6^ *	29.3 *	56.4 *	132,714 *	5.74 *	1.77 *
	Rootstock (R)	4	12,499.2 *	22.6 *	0.05 *	72,425.7 *	2.6 *	1.3 *	16,196.2 *	0.56 *	0.14 *
	Y × R	5	10,201.6 *	36.3 *	0.07 *	383,575 *	10.5 *	1.1 *	20,998.6 *	1.94 *	0.24 *
	Within	75	8779.4 ns	35.0 ns	0.01 ns	81,072.5 ns	5.3 ns	2.7 ns	2.1 × 10^5^ ns	0.56 ns	0.32 ns
	Total	89	51,772.1 ns	100.9 ns	0.16 ns	1.6 × 10^6^ ns	47.7 ns	61.5 ns	1.9 × 10^5^ ns	8.81 ns	2.45
(B)			TA	SSC	SSC:TA	TPC	TMA	AA	TPC	TMA	AA
ROOTSTOCKS	Barrier^®^		119 ± 9 a	14.3 ± 0.8 a	0.12 ± 0.02 ab	263 ± 18 b	1.7 ± 0.4 a	1.4 ± 0.2 a	101 ± 17 a	0.6 ± 0.2 a	0.5 ± 0.1 a
	Cadaman^®^		123 ± 11 a	13.2 ± 0.9 b	0.11 ± 0.02 ab	276 ± 11 b	1.9 ± 0.3 a	1.5 ± 0.3 a	81 ± 7 b	0.4 ± 0.1 a	0.5 ± 0.1 a
	GF677		131 ± 10 a	13.5 ± 0.8 b	0.10 ± 0.01 ab	282 ± 13 b	1.9 ± 0.3 a	1.5 ± 0.3 a	80 ± 6 b	0.5 ± 0.1 a	0.5 ± 0.1 a
	GxN22		115 ± 5 ab	13.2 ± 0.9 b	0.12 ± 0.02 ab	315 ± 18 a	2.0 ± 0.4 a	1.6 ± 0.2 a	81 ± 5 b	0.7 ± 0.2 a	0.5 ± 0.1 a
	Isthara^®^		96 ± 15 b	14.5 ± 0.9 a	0.17 ± 0.07 a	314 ± 19 a	1.8 ± 0.2 a	1.7 ± 0.3 a	113 ± 12 a	0.5 ± 0.1 a	0.6 ± 0.1 a
			TA	SSC	SSC:TA	TPC	TMA	AA	TPC	TMA	AA
YEARS	2011		95.9 ± 20.8 b	13.5 ± 1.2 b	0.15 ± 0.06 a	145 ± 20 b	1.3 ± 0.3 b	0.6 ± 0.3 b	76 ± 15 b	0.8 ± 0.3 a	0.7 ± 0.1 a
	2017		125.4 ± 24.1 a	14.2 ± 1.0 a	0.12 ± 0.04 a	311 ± 73 ab	2.7 ± 0.6 a	1.7 ± 0.4 a	144 ± 37 a	0.6 ± 0.1 a	0.37 ± 0.04 b
	2019		127.9 ± 15.5 a	13.6 ± 0.9 b	0.11 ± 0.02 a	412 ± 116 a	2.4 ± 0.4 a	1.7 ± 0.5 a	57 ± 13 b	0.5 ± 0.1 ab	0.23 ± 0.03 c

## Data Availability

The data used in the current study are contained within the article.
